# Risk of Introduction of Infectious Animal Diseases for Europe Based on the Health Situation of North Africa and the Arabian Peninsula

**DOI:** 10.3389/fvets.2019.00293

**Published:** 2019-09-04

**Authors:** Elena Massó Sagüés, Eduardo Fernández-Carrión, Jose Manuel Sánchez-Vizcaíno

**Affiliations:** Animal Health Department, VISAVET Health Surveillance Centre, Veterinary School, Complutense University of Madrid, Madrid, Spain

**Keywords:** risk analysis, infectious diseases, entry, vector, wind flow, birds migration, bluetongue, North Africa and Middle East

## Abstract

The current growth of the human population, the intensification of animal production, climate change or globalization favors an increase in the transmission of infectious diseases. Risk analysis is the tool that allows the identification of the factors involved in the introduction and the spread of infectious diseases. The main objective of this work is to evaluate the risk of entry of animal infectious zoonotic and non-zoonotic diseases from North Africa and the Arabian Peninsula to countries of the European Union. A probabilistic formulation has been developed to obtain the probabilities of introduction of diseases associated with each possible route of entry in the European Union. The results show that, among the infectious diseases analyzed in this study, avian influenza and Newcastle disease are the ones with a higher risk of entry in the European Union and the wild bird's migration is the route with greater impact. It is confirmed a moderate probability of entry of some vector-borne diseases, bluetongue and epizootic haemorrhagic disease, through wind flow from Morocco, Algeria and Tunisia. Due to the absence of live dromedary movement to Europe, the more likely way of entry of the Middle East respiratory syndrome is through the infected people movement from Saudi Arabia, Kuwait, Qatar and Oman. This study includes different methodologies. A model of vectors dispersion in wind currents has been established to assess the risk of introduction of vector borne diseases. It is applicable both in animal health and public health. A periodical update would be useful to obtain a periodically updated risk analysis and to allow early detection of potential hazard with an increased risk over the previous years.

## Introduction

The current growth of human population, the intensification of animal production, climate change or globalization favors an increase in the transmission of infectious diseases between distant geographical areas (1, 2). These diseases represent a threat for both human and animal health, resulting in higher mortality rates and serious economic losses with the decline in production.

The risk analysis is an important tool in epidemiology and it is essential to assess the risk of introduction and possible spread of diseases in areas with little impact (emerging and re-emerging diseases). It enables the design and implementation of control measures for the disease eradication, in order to avoid, prevent and minimize the losses or consequences arising from the transmission of diseases (3).

The development of a conceptual model is necessary to analyze the risk of introduction of diseases. It has to integrate all the identified elements that are going to intervene in the assessment of the risk. The final risk will be estimated from the evaluation of each one of the elements considered in the conceptual model, called “parameters,” and each parameter will be defined by “variables” (4, 5).

**Figure 1:**
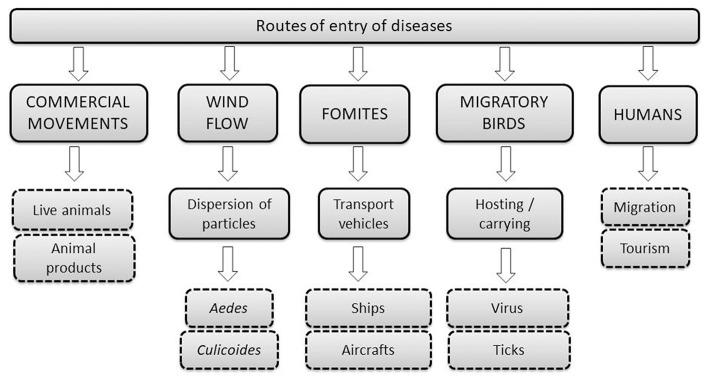
Major routes of entry of diseases from North Africa and Middle East in the European Union.

The parameters considered in this study are the different routes of entry of diseases coming from North Africa and the Arabian Peninsula into the European Union (Figure 1).

The movement of animals, both domestic and wild, is one of the main routes of entry of diseases. Animal products such as meat, milk or eggs, along with the transport of semen for artificial insemination, are possible ways of entry of pathogens (4, 5).

Wild birds are an important agent of transmission of diseases, hosting diseases such as Newcastle disease and avian influenza (4, 6), or carrying vectors like ticks that could transmit pathogens (*Coxiella, Anaplasma, Babesia, Borrelia, Rickettsia*, and tick-borne encephalitis virus) (7, 8).

The wind can be a vehicle of pathogens. Many of the epidemiological suspicions are based on the fact that certain vector-borne diseases are introduced to distant regions by the dispersion of vectors in the wind flow. It has been suggested that with a wind speed of between 10 and 40 km/h, with a temperature of between 12 and 35°C, the vector can be transported up to about 700 km (9).

Rift Valley fever, epizootic haemorrhagic diseases, lumpy skin disease and West Nile fever are examples of vector-borne diseases, transmitted by insects as *Culicoides* and mosquitoes like *Aedes*. Scientific studies prove that *Culicoides imicola*, mainly Afro-Asiatic dispersion, is spreading through Europe and it is expected a rise favored by global warming (10, 11).

There are other diseases, such as foot and mouth disease, which can also be transmitted by the wind flow (especially in warm regions) up to 60 km by land and 300 km by sea, without being a vector-borne disease (12).

The tourism and the high frequency of travels may favor an increase in the risk of transmission of diseases. Spain is the route in and out Europe. Immigrants coming from Africa and European citizens of African descent are driving frequently from Morocco to Europe across the Iberian Peninsula. We consider this entry route for diseases such as the Middle East respiratory syndrome (MERS), a zoonotic emerging disease with possible transmission within the human species. The majority of the cases have been related to healthcare centers, but transmission is possible between relatives. In an outbreak on South Korea in 2015, only one patient infected more than 70 people during his stay in a hospital emergency room for 3 days (13). According to the data provided at the WHO database (14) in January 2017, there have been 1.879 human cases of Middle East respiratory syndrome from 2012 to 2017, with 659 deaths (30–40% mortality). The 80% of the cases are contained in Saudi Arabia, but there have been MERS notifications in a total of 27 countries of Middle East, North Africa, Europe, United States and Asia (15).

Another route of entry is the movement of contaminated vehicles, potential source of transmission of diseases and can also vehicle vectors. Vehicles transporting animals that have not been correctly disinfected could be an important source of pathogens; therefore the animals that are loaded after have a high probability of infection during transport (4, 5).

Glanders is a zoonotic infectious disease, endemic in Africa. It is important to maintain the vigilance with this disease due to the importance of the horses' social network and the high number of movements for international competitions, and to control the disease to avoid its use as a biological weapon (16).

There are other possible routes of entry such as bioterrorism, biological leaks, illegal trade; but not referred to this work for not having access to detailed and concrete data for a quantitative analysis of the risk of introduction of diseases by these pathways.

The main objective of this work is to evaluate the risk of entry of animal infectious zoonotic and non-zoonotic diseases from North Africa and the Arabian Peninsula to countries of the European Union by different pathways.

## Materials and Methods

### Countries and Diseases Selection

Sixteen countries of North Africa and the Arabian Peninsula are included in this study (Table 1): Morocco, Algeria, Tunisia, Libya, Egypt, Mali, Mauritania, Niger, Chad, Sudan, United Arab Emirates, Saudi Arabia, Qatar, Oman, Kuwait, and Bahrain.

**Table 1:** Countries “i” included in the study.

**ID_**i**_**	**Abbreviations**	**Country**	**ID_**i**_**	**Abbreviations**	**Country**
1	MA	Morocco	9	ML	Mali
2	DZ	Algeria	10	MR	Mauritania
3	TN	Tunisia	11	SA	Saudi Arabia
4	LY	Libya	12	KW	Kuwait
5	EG	Egypt	13	BH	Bahrain
6	SD	Sudan	14	QA	Qatar
7	TD	Chad	15	AE	United Arab Emirates
8	NG	Niger	16	OM	Oman

Among all the infectious diseases reported in the last 12 years in the above selected countries, according to the information provided by the World Organization for Animal Health (12), 11 are selected for the analysis (Table 1): bluetongue, contagious bovine pleuropneumonia, epizootic haemorrhagic disease, foot and mouth disease, equine glanders, heartwater, highly pathogenic avian influenza, Middle East respiratory syndrome, Newcastle disease, peste des petits ruminants, and Rift Valley fever.

### Diseases Analysis and Possible Routes of Entry

The possible routes of entry of each selected disease, based on an extensive literature review, are provided (Table 2). The identification of the routes of entry has been conceptually based on the possibility of occurrence, regardless of its likelihood and frequency.

**Table 2:** Infectious diseases selected for the analysis, their identificationin this study (ID_j_), standard abbreviations, type of affected animals, mode of transmission (DC, direct contact; V, vector-born; F, fomites; I, food-borne) and routes of entry (0: no entry; 1: entry).

**ID_**j**_**	**Abbreviations**	**Disease**	**Affected animals**	**Mode of transmission**	**Animals movement**	**Animal products**	**Wild birds**	**Wind**	**People**	**Transport-vehicles**
1	BT	Blue tongue (Orbivirus)	Domestic and wild ruminants	V (*Culicoides*)	1	0	0	1	0	1
2	BCP	Bovine contagious pleuropneumonia (*Mycoplasma mycoides* subsp. *mycoides*)	Bovine	DC.	1	0	0	0	0	0
3	EHD	Epizootic haemorrhagic disease (Orbivirus)	Domestic and wild ruminants	V (*Culicoides*)	1	0	0	1	0	1
4	FMD	Foot and mouth disease (Aphtovirus)	Porcine and ruminants	DC. F. I (meat y dairy). Wind (60–300 km)	1	1	0	1	0	1
5	Gl	Glanders (*Burkholderia mallei*)	Equides Carnivores Humans	I. DC. F.	1	1	0	0	1	1
6	HW	Heartwater (*Ehrlichia ruminantium)*	Ruminants	V (tiques *Amblyomma*)	1	0	0	0	0	1
7	HPAI	Highly pathogenic avian influenza (Influenzavirus)	Avian Mammals	DC. F.	1	0	1	0	1	1
8	MERS	Middle East respiratory syndrome (MERS-CoV)	Dromedaries Humans	DC. F. I (urine, meat, dairy)	1	1	0	0	1	1
9	ND	Newcastle disease (Avulavirus)	Avian. Humans.	DC. F.	1	1	1	0	0	1
10	PPR	Peste des petits ruminants (Morbilivirus)	Small ruminants.	DC. F.	1	0	0	0	0	1
11	RVF	Rift Valley fever (Phlebovirus)	Ruminants. Humans.	V (Mosquitoes: *Aedes, Culex*)	1	1	0	1	1	1

### Data Extraction for the Analysis

The necessary data for the analysis is collected from different official databases of free access. The information relating to the movements of live animals and animal product origin between countries, are extracted from the FAOSTAT database (17) and completed with CITES Trade database (18). It provides animal movement and animal product data for imports and exports between countries. The data is obtained in quantities. To obtain information relating to the movement of vehicles and people between countries, EUROSTAT (19) database is queried. It provides flights data (origin, destiny and number of flights in a monthly basis), ships annual data (measured in tons), people international transport data, and annual migratory movements. From the OIE database WAHIS INTERFACE (20) it is extracted the data referring to animal population in each selected country, the health status for each disease of the different countries and the annual immediate notifications of each disease. Demographic information is obtained from the World Health Organization database (14).

After carrying out a literature review of the migratory areas in the world and the more frequented areas by migratory birds, “Critical Site Network Tool—Species search” (21) was used to obtain data on the species of birds present in each country and the census, and to visualize its distribution on the global map.

**Figure 2:**
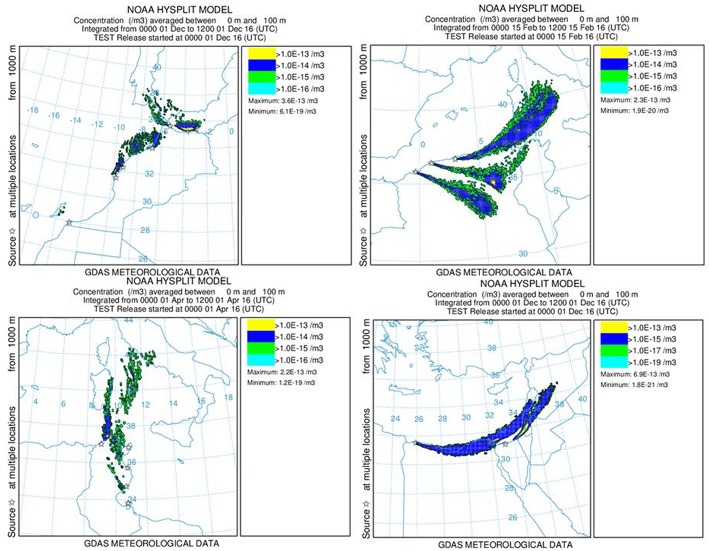
Particle dispersion models.

For the study of the possible introduction of vector borne diseases through the drag of *Culicoides* and/or mosquitoes by wind currents, simulations (Figure 2) are made for the estimation of wind and particle's dispersion trajectories that reached the European territory during the year 2016. The program “*HYSPLIT—Hybrid Single Particle Lagrangian Integrated Trajectory model*” (22) was used to locate areas and periods of maximum diffusion of particles. This program, facilitated by U.S. National Oceanic and Atmospheric Administration's Air Resources Laboratory (NOAA-ARL), allows creating simulations in any geographical coordinate of the globe, to different heights and dates, by using a file of GDAS (Global Data Assimilation System) climate data. This model has been used in other studies of dispersion of arthropods, especially of *Culicoides* as carriers of bluetongue (9, 23).

The program allows the creation of dispersion models for different densities (ρ) and particles size. Densities of 0.28 g/cc for *Culicoides* and 0.06 g/cc for *Aedes aegypti* has been obtained using the formula ρ = m/V, *m* mass and *v* volume. The following data has been used for this calculation: *Culicoides* with 1.5 mm size and 0.5 mg mass (10), and *Aedes aegypti* with 4 mm size and 3 mg mass (24).

### Analysis of the Risk of Entry Based on Probability

Our interest is to calculate the probability of occurrence of specific events: the probability of entry of ***j*** diseases by different routes from country ***i***; where, henceforth, **i∈{1, …, 16}** and **j ∈{1, …, 11}**. The probability of occurrence of an event A, ***p***(***A***), is defined as the quotient between the number of favorable cases to the event A and the number of possible cases (3, 4):

(1) $$\begin{array}{r} p\left(A\right)\;=\;\frac{number\;of\;favorable\;cases\;to\;the\;event\;A}{number\;of\;possible\;cases} \end{array}$$

The different probabilities calculated in this study are named as different variables:

${PIA}_{i}^{j}$–Probability of introduction of the ***j*** disease into the European Union through live animals trade from the country **i**.
${PIT}_{i}^{j}$–Probability of introduction of the ***j*** disease into the European Union through the movement of transport vehicles from the country ***i***.
${PIP}_{i}^{j}$–Probability of introduction of the ***j*** disease into the European Union through the movement of people from the country ***i***.
${PIS}_{i}^{j}$–Probability of introduction of the ***j*** disease into the European Union through the importation of animal products from the country ***i***.
${PIM}_{i}^{j}$–Probability of introduction of the ***j*** disease into the European Union through the movement of migratory birds from the country ***i***.
${PIV}_{i}^{j}$–Probability of introduction of the ***j*** disease into the European Union through the dispersion of particles in wind currents from the country ***i***.

Each probability is calculated as the probability of a set of independent events (*X*1, *X*2…), being the first event the probability of infection in the country of origin and the last one the likelihood of introduction into the country of destination.

(2) $$\begin{array}{r} PIX\;=\;P\left(X1\cap X2\cap \ldots \right)=P\left(X1\right)▪P\left(X2\right)▪\ldots \end{array}$$

The first event is the probability of the country **i** being affected by the disease **j**, named as ${PPA}_{i}^{j},\;$ calculated as an average based on the number of outbreaks notified in the last 12 years (from 2005 to 2017). We used a weighted moving average formula that gives a high probability when the country of origin had notified outbreaks in the last few years (2017, 2016, 2015 …) and a lower probability to those countries that had notified outbreaks in the first years included in the study (2005, 2006, 2007 …). Therefore, in the extreme cases, when a disease has never been notified or, conversely, it has been endemic in a country during the period of study, the probability will be zero or one, respectively.

The formula that is needed for the estimation of the likelihood of a country **i** being affected by the diseases **j** is:

$$\begin{array}{l} {PPA}_{i}^{j}\;=\;\frac{B_{i,2005}^{j}\times 1+B_{i,2006}^{j}\times 2+B_{i,2007}^{j}\times 3+\ldots +B_{i,2015}^{j}\times 11+B_{i,2016}^{j}\times 12}{1+2+3+\ldots +11+12} \end{array}$$

The $B_{i,20XX}^{j}$ represents the binary value **0/1** –The value 1 is associated to the occurrence of an outbreak of the disease **j** on the country **i** in the year 20XX. The results are shown in Table 3.

**Table 3:** Probability of the country **i** being affected by the disease **j** $\left(PPA_{i}^{j}\right)$.

**PPA**		**Disease “j”**										
**Country “i”**		**1**	**2**	**3**	**4**	**5**	**6**	**7**	**8**	**9**	**10**	**11**
		**BT**	**BCP**	**EHD**	**FMD**	**Gl**	**HW**	**HPAI**	**MERS**	**ND**	**PPR**	**RVF**
1	MA	0,17	0	0,03	0,14			0			0,19	0
2	DZ	0,31	0	0,03	0,27	0	0	0,15		0,13	0,46	0
3	TN	0,35	0	0,17	0,13	0	0	0,15		0,15	0,35	0
4	LY	0,14			0,26			0,27		0,25	0,14	
5	EG	0	0	0	0,24	0	0	0,18			0,25	0
6	SD	0		0	0,15	0	0,15	0,03		0,15	0,15	0,09
7	TD	0	0,15	0	0,15		0,08			0,15	0,15	0
8	NG	0	0,15	0	0,15	0		0,32		0,15	0,15	0,15
9	ML		0,15	0	0,15			0			0,16	0,15
10	MR	0	0,27		0,14						0,15	0,49
11	SA	0,15		0	0,29	0		0,04	0,15	0,15	0,15	0,3
12	KW		0	0	0,22		0	0,21	0,13	0,15	0,15	0
13	BH	0	0	0	0,12	0,17	0	0			0	0
14	QA	0,08	0	0	0,15			0	0,12	0,15	0	0
15	AE	0	0	0	0,06	0	0	0			0,15	0
16	OM	0,06	0	0	0,14	0	0	0	0,13	0,14	0,14	0

*There is no available information about some diseases in some countries; therefore, no probability has been assigned for those countries. In addition, for the countries that had never notified a disease, it has been assigned a zero probability*.

### Description of Each Probability Calculated

#### Probability of Introduction Through Live Animals Trade

The probability of introduction of the **j** disease into the European Union through live animal's trade from the country ***i*** $\left(PIA_{j}^{i}\right)$ is calculated as the proportion of animals that are annually transported to Europe coming from the country **i** multiplied by the probability of the country **i** being affected by the disease ***j*** $\left(PPA_{i}^{j}\right)$.

$$\begin{array}{l} {PIA}_{i}^{j}\;=\;\frac{animals\;transported\;to\;Europe\;from\;the\;country\;i}{animal\;population\;in\;the\;country\;i}▪PPA_{i}^{j} \end{array}$$

#### Probability of Introduction Through Transport-Vehicles

The formula chosen to estimate the probability that a **j** disease reaches the European Union coming from the country **i** through transport-vehicles $\left(PIT_{j}^{i}\right)$ is a ratio between the vehicles or load transported arriving to the European Union from the country **i** and the total vehicles or load transported to the European Union, multiplied by the probability of the country **i** being affected by the disease ***j*** $\left(PPA_{i}^{j}\right)$ and the probability of the vehicles being contaminated by the pathogen responsible for the disease **j**.

The probability of a vehicle being contaminated is obtained from the average survival of the pathogen in surfaces (***PSA***_***j***_) and the average disinfection of vehicles (**0.1**). It is estimated that one out of every ten vehicles cannot be effectively disinfected allowing the survival of pathogens (5). The average survival of pathogens in surfaces (***PSA***_***j***_) is obtained based in the literature review (25) and available data. Glanders, Newcastle disease and heartwater infective pathogens have a long average survival, more than 60 days (***PSA***_***j***_ = 1). Foot and mouth disease and highly pathogenic avian influenza infective pathogens have an intermediate average survival, around 30 days (***PSA***_**4**_ = 0.47; ***PSA***_**7**_ = 0.39). Peste des petits ruminants and Middle East Respiratory Syndrome infective pathogen have a short average survival, around 4 days (***PSA***_***j***_ = 0.06).

$$\begin{array}{l} PIT_{i}^{j}\;=\;\left(\frac{load\;transported\;by\;sea\;from\;i\;to\;EU}{total\;load\;transported\;to\;EU}\;x\;PSA_{j}x\;0,1\;x\;PPA_{i}^{j}\right)\\ \;+\left(\frac{n^{\underline{\text{o}}}\;aircrafts\;from\;i\;to\;EU}{total\;n^{\underline{\text{o}}}\;of\;aircrafts\;to\;EU}\;x\;PSA_{j}x\;0,1\;x\;PPA_{i}^{j}\right) \end{array}$$

#### Probability of Introduction Through the Movement of People

The probability of introduction of the **j** disease into the European Union through the movement of people from the country ***i*** $\left(PIP_{j}^{i}\right)$ is calculated as the intersection of two events: the probability of the country **i** being affected by the disease ***j*** $\left(PPA_{i}^{j}\right)$ and the probability of a person from the country **i** arriving to the European Union $\left(PHE_{i}^{j}\right)$.

$$\begin{array}{l} {PIP}_{i}^{j}\;=\;{PHE}_{i}x\;{PPA}_{i}^{j} \end{array}$$

The probability of a person from the country **i** arriving to the European Union $\left(PHE_{i}^{j}\right)$. is the ratio between the people coming to the European Union from the country **i** and the total human population in the country **i**.

$$\begin{array}{l} {PHE}_{i}\;=\;\frac{airline\;passengers+ship\;passengers+immigrants\;from\;i\;}{human\;population\;on\;the\;country\;i} \end{array}$$

#### Probability of Introduction Through the Importation of Animal Products

The probability of introduction of the **j** disease into the European Union through the importation of animal products from the country ***i*** $\left(PIS_{j}^{i}\right)$ is the ratio between the proportion of animal products that are annually transported to the European Union coming from the country **i** and the total animal products transported to the European Union, multiplied by the probability of the country **i** being affected by the disease ***j*** $\left(PPA_{i}^{j}\right)$.

$$\begin{array}{l} {PIS}_{i}^{j}=\frac{animal\;product\;imported\;form\;coutnry\;i\;to\;EU\;}{total\;animal\;products\;imported\;to\;EU}\;x\;{PPA}_{i}^{j} \end{array}$$

#### Probability of Introduction Through the Movement of Migratory Birds

The probability of introduction of the **j** disease into the European Union through the movement of migratory birds from country ***i*** $\left(PIM_{j}^{i}\right)$ is calculated as the proportion of the migratory birds population in the country **i** multiplied by the probability of the country **i** being affected by the disease **j** $\left(PPA_{i}^{j}\right)$, taking into account only the bird-borne diseases.

$$\begin{array}{l} {PIM}_{i}^{j}\;=\;\frac{migratory\;birds\;population\;in\;country\;i\;}{total\;birds\;population\;in\;country\;i\;}\;x\;{PPA}_{i}^{j} \end{array}$$

#### Probability of Introduction Through Dispersion of Particles in Wind Currents

It has been carried out simulations of the 365 days of the year 2016 for two sizes of particles: a 4 mm diameter and 0.068 g/cc density for *Aedes*, and a 1.5 mm diameter and 0.28 g/cc density for *Culicoides*.

The probability of introduction of the **j** disease into the European Union through the dispersion of particles in wind currents from the country ***i*** $\left(PIV_{i}^{j}\right)$ is calculated as the average of the number of wind simulations for each country **i** that reaches European areas, multiplied by the probability of the country **i** being affected by the disease ***j*** $\left(PPA_{i}^{j}\right)$ and the probability of the vector or infective particle remaining viable during the route$\left(PSV_{i}^{j}\right)$.

$$PIV_{i}^{j}\;=\;\frac{n^{\underline{\text{o}}}\;of\;days\;the\;dispersion\;reaches\;the\;EU}{365\;days}\;x\;PPA_{i}^{j}x\;PSV_{i}^{j}$$

The probability of the vector or infective particle remaining viable during the route $\left(PSV_{i}^{j}\right)$ is established with a review of the conditions of rainfall and humidity in the countries of study (Weather History|Weather Underground). According to the sources consulted, the foot and mouth disease virus needs specific conditions for the dispersal over long distances by air and its survival is determined by the relative humidity, below 55 per cent the virus is inactivated (26). For mosquitos and *Culicoides*, above 40°C temperature and below 14°C temperature, their survival is limited (27).

A zero probability is assigned when the weather conditions do not allow the survival of the vector or infective particle, and a probability one is assigned when the conditions are optimal for their survival and dispersion.

$$PSV_{i}^{j}\;=\;\frac{n^{\underline{\text{o}}}\;days\;with\;favorable\;weather\;conditions}{30\;days}$$

### Total Probability of Diseases Entry

With all the probabilities calculated already, we can calculate the total probability of entry of the disease **j** from the country **i** to the European Union, taking into account all the routes of entry already evaluated$\left(PI_{i}^{j}\right)$.

To do this, we calculate the probability of occurrence of the opposite case, the probability of no introduction of the **j** disease by any of the routes of entry, using the following formula:

$$\begin{array}{l} PI_{i}^{j}=1-[\left(1-PIA_{i}^{j}\right)\cdot \left(1-PIT_{i}^{j}\right)\cdot \left(1-PIP_{i}^{j}\right)\cdot \left(1-PIS_{i}^{j}\right)\\ \;\cdot \left(1-PIM_{i}^{j}\right)\cdot \left(1-PIV_{i}^{j}\right)] \end{array}$$

With the same type of formula, it is estimated the likelihood of entry of a disease **j** in the European Union.

$$PI_{j}\;=\;1-\prod_{i}\left(1-PI_{i}^{j}\right)$$

A high, moderate and low risk of introduction of infectious diseases from different countries has been estimated based on a 75 and 90-percentile (P_75_ and P_90_) over the final results of probability of each route of entry. Therefore, the results that are over the 75-percentile and 90-percentile are classified as moderate and high risk of entry.

### Validation of the Model

For the validation of the model, historic data has been used to relate the obtained results with the historic events. The information available in the OIE database WAHIS INTERFACE (20) has been used to the extraction of the data referring to the exceptional epidemiological events in the European Union countries during the last 2 years (2017 and 2018).

## Results

Following the methodology described in the previous section quantitative probabilities were obtained. The probabilities of entry for each route, country and disease are detailed in the following tables: Tables 4–9. There are some countries without information available regarding certain diseases, therefore, no probability has been assigned.

**Table 4:** Probability of introduction of the **j** disease into the European Union through live animal's trade from the country **i**.

**PIA**		**Disease “j”**								
**Country “i”**		**1**	**2**	**3**	**4**	**5**	**6**	**7**	**9**	**11**
		**BT**	**BCP**	**EHD**	**FMD**	**Gl**	**HW**	**HPAI**	**ND**	**RVF**
1	MA	0	0	0	0		0	0	0	0
2	DZ	0	0	0	0	0	0	0	0	0
3	TN	7.7E-5	0	3.7E-5	2.8E-5	0	0	0	0	0
5	EG	0	0	0	0	0	0	5.5E-10		0
9	ML	0	0	0	0	0	0	0		0
11	SA	7.6E-6		0	1.4E-5	0		1.2E-9	4.6E-9	1.5E-5
12	KW	0	0	0	0		0	0	0	0
13	BH	0	0	0	0	4.1E-4	0	0	0	0
14	QA	0	0	0	0		0	0	1.7E-7	0
15	AE	0	0	0	8.9E-4	0	0	0		0
16	OM	1.6E-7	0	0	3.7E-7	0	0	0	0	0

*There is no available information about some diseases in some countries; therefore, no probability has been assigned for those countries. In addition, for the countries that had never notified a disease, it has been assigned a zero probability*.

**Table 5:** Probability of introduction of the **j** disease into the European Union through transport-vehicle from the country **i**.

**PIT**		**Disease “j”**									
**Country “i”**		**1**	**3**	**4**	**5**	**6**	**7**	**8**	**9**	**10**	**11**
		**BT**	**EHD**	**FMD**	**Gl**	**HW**	**HPAI**	**MERS**	**ND**	**PPR**	**RVF**
1	MA	1.7E-3	2.9E-4	6.78E-4			0		0	1.2E-4	0
2	DZ	6.5E-3	6.3E-4	2.68E-3	0	0	1.4E-3		2.7E-3	6.1E-4	0
3	TN	1.9E-3	9.6E-4	3.48E-4	0	0	3.3E-4		8.4E-4	1.2E-4	0
4	LY	1.7E-3		1.49E-3			1.3E-3		3.01E-3	1.07E-4	
5	EG	0	0	4.06E-3	0	0	2.5E-3			5.6E-4	0
6	SD	0	0	3.66E-5	0	7.7E-5	6.1E-6		7.7E-5	4.9E-6	4.6E-5
8	NG	0	0	3.78E-5	0		6.7E-5		7.9E-5	5.04E-6	7.9E-5
9	ML		0	2.36E-5			0			3.4E-6	4.9E-5
10	MR	0		1.76E-4						2.5E-5	1.3E-3
11	SA	2.7E-3	0	2.46E-3	0		2.8E-4	1.7E-4	2.7E-3	1.7E-4	5.3E-3
12	KW		0	4.88E-4		0	3.9E-4	3.8E-5	7.0E-4	4.4E-5	0
13	BH	0	0	2.4E-4	7.1E-4	0	0			0	0
14	QA	1.4E-3	0	1.29E-3			0	1.4E-4	2.7–3	0	0
15	AE	0	0	5.01E-4	0	0	0			1.7E-4	0
16	OM	1.07E-4	0	1.19E-4	0	0	0	1.5E-5	2.5E-4	1.6E-5	0

*There is no available information about some diseases in some countries; therefore, no probability has been assigned for those countries. In addition, for the countries that had never notified a disease, it has been assigned a zero probability*.

**Table 6:** Probability of introduction of the **j** disease into the European Union through people movement from the country **i**.

**PIP**		**Disease “j”**			
**Country “i”**		**5**	**7**	**8**	**11**
		**Gl**	**HPAI**	**MERS**	**RVF**
1	MA		0		0
2	DZ	0	2.94E-5		0
3	TN	0	4.21E-5		0
4	LY		4.64E-5		
5	EG	0	1.08E-5		0
6	SD	0	8.72E-7		2.61E-6
7	TD				0
8	NG	0	6.03E-6		2.82E-6
9	ML		0		2.7E-6
10	MR				1.24E-4
11	SA	0	2.57E-6	9.66E-6	1.93E-5
12	KW		5.14E-5	3.18E-5	0
13	BH	6.22E-6	0		0
14	QA	2.29E-5	0		0
15	AE	0	0		0
16	OM	0	0	7.66E-6	0

*There is no available information about some diseases in some countries; therefore, no probability has been assigned for those countries. In addition, for the countries that had never notified a disease, it has been assigned a zero probability*.

**Table 7:** Probability of introduction of the **j** disease into the European Union through importation of animal products from the country **i**.

**PIS**		**Disease “j”**				
**Country “i”**		**4**	**5**	**8**	**9**	**11**
		**FMD**	**Gl**	**MERS**	**ND**	**RVF**
1	MA	1.75E-3	0		0	
2	DZ	0	0	0	9.98E-3	0
3	TN	2.44E-4	0		0	0
5	EG	1.03E-2	0			0
8	NG	1.11E-2	0		0	1.11E-2
10	MR	9.43E-2			0	0
11	SA	7.27E-3	0	3.76E-3	0	2E-3
12	KW	6.89E-4	0	3.66E-4		0
13	BH	4.13E-4	0		1.59E-4	0

*There is no available information about some diseases in some countries; therefore, no probability has been assigned for those countries. In addition, for the countries that had never notified a disease, it has been assigned a zero probability*.

**Table 8:** Probability of introduction of the **j** disease into the European Union through wild birds migration from the country **i**.

**PIM**		**Disease “j”**	
**Country “i”**		**7**	**9**
		**HPAI**	**ND**
1	MA	0	0
2	DZ	0.145	0.125
3	TN	0.146	0.146
4	LY	0.257	0.238
5	EG	0.152	0
6	SD	1.57E-2	7.85E-2
7	TD	0	0.108
8	NG	0.239	0.112
9	ML	0	0
10	MR	0	0
11	SA	2.82E-2	0.105
12	KW	0.142	0.101
13	BH	0	0
14	QA	0	9.19E-2
15	AE	0	0
16	OM	0	7.77E-2

**Table 9:** Probability of introduction of the **j** disease into the European Union through dispersion of particles in wind currents coming from the country **i**.

**PIV**		**Disease “j”**			
**Country “i”**		**1**	**3**	**4**	**11**
		**BT**	**EHD**	**FMD**	**RVF**
1	MA	8.5E-2	1.5E-2	2.25E-3	0
2	DZ	2.58E-2	2.5E-3	2.25E-3	0
3	TN	0.116	5.66E-2	6.9E-3	0
4	LY	2.33E-2		0	
5	EG	0	0	0	0
6	SD	0	0	0	0
7	TD	0	0	0	0
8	NG	0	0	0	0
9	ML	0	0	0	0
10	MR	0	0	0	0
11	SA	0	0	0	0
12	KW	0	0	0	0
13	BH	0	0	0	0
14	QA	0	0	0	0
15	AE	0	0	0	0
16	OM	0	0	0	0

*There is no available information about some diseases in some countries; therefore, no probability has been assigned for those countries. In addition, for the countries that had never notified a disease, it has been assigned a zero probability*.

**Figure 3:**
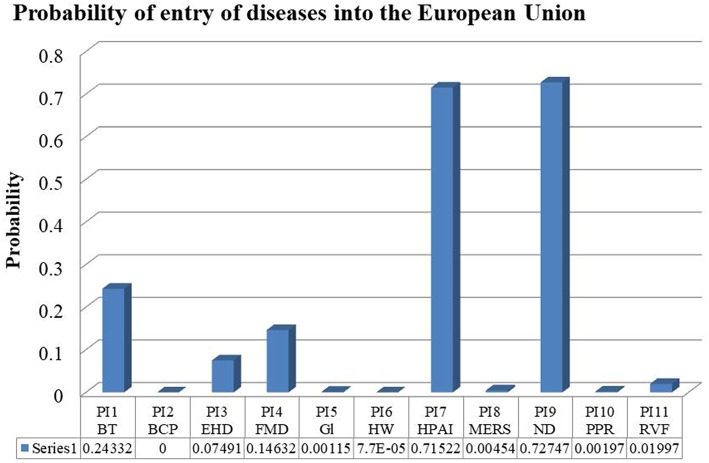
Likelihood of entry of each disease in the European Union (PI: Probability of Introduction). Disease 1: bluetongue; 2: bovine contagious pleuropneumonia; 3: epizootic haemorrhagic disease; 4: foot and mouth disease; 5: glanders; 6: heartwater; 7: highly pathogenic avian influenza; 8: Middle East respiratory syndrome; 9: Newcastle disease; 10: peste des petits ruminants; 11: Rift Valle fever.

With all the probabilities already calculated, the total probability of entry of the disease **j** from the country **i** into the European Union by any combination of routes of entry $\left(PI_{i}^{j}\right)$ is obtained and detailed in Table 10. Furthermore, the likelihood of entry of a disease **j** in the European Union is represented in Figure 3.

**Table 10:** Total probability of entry of the **j** disease from the country **i** to the European Union.

**PIij**		**Disease “j”**										
**Country “i”**		**1**	**2**	**3**	**4**	**5**	**6**	**7**	**8**	**9**	**10**	**11**
		**BT**	**BCP**	**EHD**	**FMD**	**Gl**	**HW**	**HPAI**	**MERS**	**ND**	**PPR**	**RVF**
1	MA	8.6E-2	0	1.5E-2	4.8E-3	0	0	0	0	0	1.2 E-4	0
2	DZ	3.2E-2	0	3.1E-3	4.8E-3	0	0	1.5E-1	0	1.4E-1	6.1E-4	0
3	TN	1.2E-1	0	5.8E-2	7.5E-3	0	0	1.5E-1	0	1.5E-1	1.3E-4	0
4	LY	2.5E-2	0	0	1.5E-3	0	0	2.6E-1	0	2.4E-1	1.1E-4	0
5	EG	0	0	0	1.1E-2	0	0	1.5E-1	0	0	5.6E-4	0
6	SD	0	0	0	3.7E-05	0	7.7E-5	1.6E-2	0	7.8E-2	4.9E-6	4.9E-5
7	TD	0	0	0	0	0	0	0	0	1.1E-1	0	0
8	NG	0	0	0	1.1E-2	0	0	2.4E-1	0	1.1E-1	5.0E-6	1.1E-2
9	ML	0	0	0	2.4E-05	0	0	0	0	0	3.4E-6	5.2E-5
10	MR	0	0	0	9.4E-2	0	0	0	0	0	2.5E-5	1.4E-3
11	SA	2.7E-3	0	0	9.7E-3	0	0	2.8E-2	3.9E-3	1.1E-1	1.7E-4	7.4E-3
12	KW	0	0	0	1.8E-3	0	0	1.4E-1	4.4E-4	1.0E-1	4.4E-5	0
13	BH	0	0	0	6.5 E-4	1.1E-3	0	0	0	1.6E-4	0	0
14	QA	1.4E-3	0	0	1.3E-3	2.3E-5	0	0	1.4E-4	9.4E-2	0	0
15	AE	0	0	0	1.4E-3	0	0	0	0	0	1.7E-4	0
16	OM	1.1E-4	0	0	1.2 E-4	0	0	0	2.2E-5	7.8E-2	1.6E-5	0

The results show that contagious bovine pleuropneumonia has no risk of entry. This disease is not only absent in Europe at the moment, but it is only transmissible by direct contact between cattle, and according to the databases consulted, the movement of cattle among the affected countries and the European Union is not made.

On the other hand, highly pathogenic avian influenza and Newcastle disease have a major risk of entry into the European Union. These disease have a high risk of entry into the European Union through wild birds migration, due to the existence of different migratory routes that link the European breeding areas with African wintering areas (7, 8), resulting in a constant flow of wild birds among the affected countries of Africa and the Arabian Peninsula, and the European Union.

Bluetongue, epizootic haemorrhagic disease, foot and mouth disease and Rift Valle fever resulted to have a moderate risk of entry into the European Union. It has been demonstrated the possibility of entry of this diseases into the European Union through wind dispersion of vectors for bluetongue, epizootic haemorrhagic disease and Rift Valley fever; or through wind dispersion of virus for foot and mouth disease.

A low risk of entry has been obtained for the following diseases: Middle East respiratory syndrome, peste des petits ruminants, glanders and heartwater.

To complete the study, the results obtained are compared with the historic events occurred during the last 2 years, shown in Table 11. In 2017 and 2018, European countries had notified outbreaks of bluetongue, highly pathogenic avian influenza, Newcastle disease and Rift Valley fever (among all the diseases included in this study).

**Table 11:** Diseases outbreaks in the European countries during the years: 2017 and 2018 (OIE World Animal Health Information System).

**2017 and 2018 Outbreak's**	**Disease “j”**			
**EU country**	**1**	**7**	**9**	**11**
	**BT**	**HPAI**	**ND**	**RVF**
Belgium		18		
Bulgaria		7	4	
Croatia		12		
Cyprus		1	1	
Czech Republic		72	1	
Denmark		22		
France			1	1
Germany		10		
Greece	9	7		12
Hungary				
Italy	1	98		
Luxembourg		4		
Netherlands		14		
Poland		2		
Portugal		1	2	1
Romania			8	
Slovakia		61		
Slovenia		21		
Spain		12		1
Sweden		9	3	
Switzerland	2	8	1	
Ukraine		5		
United Kingdom		25		
Total	12	366	19	14

## Discussion

This work demonstrates the possibility of assessing the risk of entry of different infectious diseases at the same time, and by different routes of entry into a large geographical area. Different methods has been used; some of them already deployed in other studies about one single disease, African swine disease (5), avian influenza (6), bluetongue (9), etc. In particular, the dispersion of particles in wind currents model has proved to be a very useful tool for the analysis of risk of spreading of certain vector borne diseases.

It is applicable both in animal health and in public health, in the interest to improve the health and well-being through the prevention of risk and mitigation of the effects of emerging diseases that originate from the interface between humans, animals and the natural environment (28). Once the model is complete, an annual review may be useful to update the variables and parameters that are being used and to obtain a periodically updated risk analysis which allows detecting potential hazard with an increased risk over the previous years.

In this study we worked with a few parameters and variables that can be subject to improvement. It is possible to increase the number of diseases studied as well as the routes of entry included in this model. It would be interesting, with the necessary data, to include the illegal trade as another possible way of entry. We have evaluated the risk of introduction, not the risk of spread of disease, it may also be interesting to add the probability of the infectious agent that has been introduced, to come into contact with sensitive populations and disseminate.

### Results Analysis

With the obtained results, the following diseases are not currently presenting a risk of entry through animal trade: heartwater, bovine contagious peripneumonia, Middle East respiratory syndrome, and peste des petits ruminants. Glanders has no risk of introduction through equine meat, although it has risk of entry through horse trade from Bahrain and through people and transport vehicles movement (as fomites). Rift Valley fever has no risk of entry in the European Union through wind dispersion of viruses, although it has a certain risk of entry through animal and dairy products trade, and transport vehicles movement containing vectors of the disease.

#### Animal and Animal Products Trade

The probability of entry of infectious diseases through animal and animal products trade is relatively low, due to the shortage of commercial movements between these countries and the European Union (see Supplementary Material).

In the movement of live animals, Tunisia represents a greater risk of entry of bluetongue and epizootic haemorrhagic disease for the European Union, Bahrain of glanders and United Arab Emirates of foot and mouth disease. There is a lower risk of entry of highly pathogenic avian influenza through the movement of live birds from Egypt and Saudi Arabia.

In the movement of animal products, the countries representing a higher risk of entry of diseases are Egypt, Mauritania, Niger and Saudi Arabia, the three of them with a higher risk of introduction of foot and mouth disease into the European Union.

#### People Movement and Transport-Vehicles

The probability of entry of infectious diseases through people movement and transport-vehicles is low, but they are the only likely routes of entry of certain diseases such as heartwater, peste des petits ruminants, and Middle East respiratory syndrome (MERS).

Cowdriosis is a vector-borne disease transmitted by ticks (*Amblyoma* genus in the majority of cases). The only likely route of entry of this disease into the European Union is through the movement of infected animals or carriers of the tick (with a cero probability obtained) of through the transport of the infected tick in transport-vehicles.

The most likely entrance of the Middle East respiratory syndrome in the European Union is through infected people movement (travelers of immigrants) from countries of the Arabian Peninsula. Outbreaks of this disease had occurred in Europe in 2016, the origin was a person coming from Saudi Arabia (14).

The peste des petits ruminants, transmissible by direct contact and fomites, represents a risk for Europe only by the movement of vehicles contaminated by previous trips in which carrying this type of cattle.

#### Wild Bird Migration

The probability of introduction of highly pathogenic avian influenza and Newcastle disease through wild bird migration is high due to the existence of different migratory routes that link the European breeding areas with African wintering areas (7, 8), resulting in a constant flow of wild birds among the affected countries of Africa and the Arabian Peninsula, and the European Union.

This pathway may be responsible for the recent entry of Newcastle disease in the European Union, in the last 2 years (2017 and 2018) outbreaks of Newcastle disease has been notified in Portugal, France, Rumania, Bulgaria, Sweden, Switzerland, Check Republic and Cyprus (Table 11). The origin of the outbreaks is not defined but following the results obtained, it may be possible that a percentage of these outbreaks may be caused by wild bird's migration from African and Middle East wintering areas.

#### Dispersion of Particles in Wind Currents

There is a high risk of introduction of bluetongue and epizootic haemorrhagic disease in the European Union through dispersion of particles in wind currents from Morocco, Algeria, Tunisia and Libya, and a moderate risk of introduction of foot and mouth disease through this pathway. The result of bluetongue is validated by the recent notifications; in the last 2 years (2017 and 2018) outbreaks of bluetongue have been notified in Greece, Italy, and Switzerland (Table 11).

In the other hand, there is no risk of entry of Rift Valley fever through this pathway. If the North African Coast countries, the countries of the study that show in the simulations a certain probability of particles dispersion arriving to the European Union, remain free of Rift Valley fever, there will be no risk of introduction of this disease.

To confirm the absence of risk of introduction of Rift Valley fever through this pathway, the situation of the disease in the countries of the study, which so far are not affected, should be checked periodically.

### Limitations of the Study

The main limitation of the model is the information available. The majority of information managed in this study is coming from official databases (PubMed, OIE 18, GDAS 25, EUROSTAT 22, FAOSTAT 21), although they do not have information from all countries of interest. There is only complete information of five countries: Morocco, Algeria, Tunisia, Egypt, and Saudi Arabia.

There is not enough information for certain emerging diseases such as Middle East respiratory syndrome, so this study can be updated as the research on the different diseases transmission routes progresses.

In the same way, there are countries without information available, for lack of declaration on the current health situation or underreporting of diseases outbreaks.

Once the model is complete, could conduct an annual or biannual review to update the variables and parameters in use, to obtain an updated risk analysis that allows detecting potential dangerous routes of entry by an increase in the risk from the previous period.

## Conclusions

This work has made possible to assess the risk of entry of different infectious diseases at the same time, and through different routes of entry into a large geographical area.

The use of spread sheets for the development of probabilistic formulation has been of vital importance for the collection and analysis of data, although its validity depends on the confidence and quality of the available information. In this case, there is only complete information of five countries: Morocco, Algeria, Tunisia, Egypt, and Saudi Arabia.

It has been established a model for vectors introduction in wind flow that confirms the potential entry by this pathway of some vector-borne diseases, bluetongue and epizootic haemorrhagic disease, from Morocco, Algeria and Tunisia.

Of all the diseases analyzed in this study, Newcastle disease and avian influenza are the ones with a higher risk of entry in the European Union. The pathway with more relevance in the risk of entry of these diseases is the wild bird's migration.

The diseases with a moderate risk of entry are bluetongue, epizootic haemorrhagic disease and foot and mouth disease. These diseases have in common the possible entry through wind dispersion. In the case of vector-borne diseases it is possible by vectors dispersion in wind currents, and in the case of foot and mouth disease it is possible by virus spreading through wind currents.

Due to the absence of live dromedary movement to Europe, the more likely way of entry of the Middle East respiratory syndrome is through infected people movement, from Saudi Arabia, Kuwait, Qatar and Oman.

The contagious bovine pleuropneumonia is the only disease with no risk of introduction in the European Union, due to the absence of cattle movement from the countries affected by this disease, Chad, Niger, Mali, and Mauritania.

## Data Availability

Publicly available datasets were analyzed in this study. This data can be found here: http://www.fao.org/faostat/en/#data/TA, http://www.fao.org/faostat/en/#data/TP, https://ec.europa.eu/eurostat/data/database, http://www.oie.int/wahis_2/public/wahid.php/Wahidhome/Home, http://apps.who.int/gho/data/node.country, http://csntool.wingsoverwetlands.org/csn/default.html#state=speciesSearch, not available any more and changed into http://critical-sites.wetlands.org/en, http://ready.arl.noaa.gov/HYSPLIT.php.

## Author Contributions

JS-V conceived the idea for the work. EM and EF-C designed the work. EM carried out the data extraction for the risk analysis. EM and EF-C developed the probabilistic formulation. EM designed the tables with the results for interpretation. EF-C contributed in the interpretation of the program HYSPLIT. EM obtained the wind and particle dispersion simulations and estimated trajectories. EM obtained data from Critical Site Network Tool—Species search and studied species distribution in the global map. JS-V reviewed the design of the work and the results obtained. EM wrote the manuscript. EF-C and JS-V provided critical feedback and helped shape the research, analysis, and manuscript.

### Conflict of Interest Statement

The authors declare that the research was conducted in the absence of any commercial or financial relationships that could be construed as a potential conflict of interest.
