# Supplementary material for: Risk of Introduction of Infectious Animal Diseases for Europe Based on the Health Situation of North Africa and the Arabian Peninsula
Source: Front Vet Sci. 2019 Sep 4;6:293. doi: 10.3389/fvets.2019.00293 (PMC6737002; doi:10.3389/fvets.2019.00293)
Supplement: Supplementary file 1 [file Data_Sheet_1.docx]

Supplementary Material

**Risk of introduction of infectious animal diseases for Europe based on the health situation of North Africa and the Arabian Peninsula**

**Massó Sagüés, E*., Fernández-Carrión, E. y Sánchez-Vizcaíno J.M.**

*** Correspondence:** Elena Massó Sagüés: [elenamassosagues@gmail.com](mailto:elenamassosagues@gmail.com)

# Supplementary Data

**Disease outbreaks**. Sheet 1 includes all the diseases outbreaks in the countries included in the study from 2005 to 2016. Sheet 2 includes only the outbreaks for the diseases included in the study for the countries included in the study from 2005 to 2016.

**Wild bird census**. Sheet 2 includes the population of birds species, expressed in thousands of birds, that migrate between the countries of the Arabian Peninsula, North Africa and the European Union (Critical Site Network Tool - Species search).

**Air transport graphic**. International freight flights, expressed in number of flights, between the countries of the European Union and the countries included in the study (EUROSTAT).

**Maritime transport graphic**. Gross weight of goods, expressed in tones, transported in vessels from the countries included in the study to the European Union (EUROSTAT). There is no information available for the following countries: Chad (number 7) and Mali (number 9).

**Immigration graphic**. Annual migration to the European Union, expressed in number of people, depending on their country of origin (EUROSTAT).

# Supplementary Figures and Tables

## Supplementary Figures


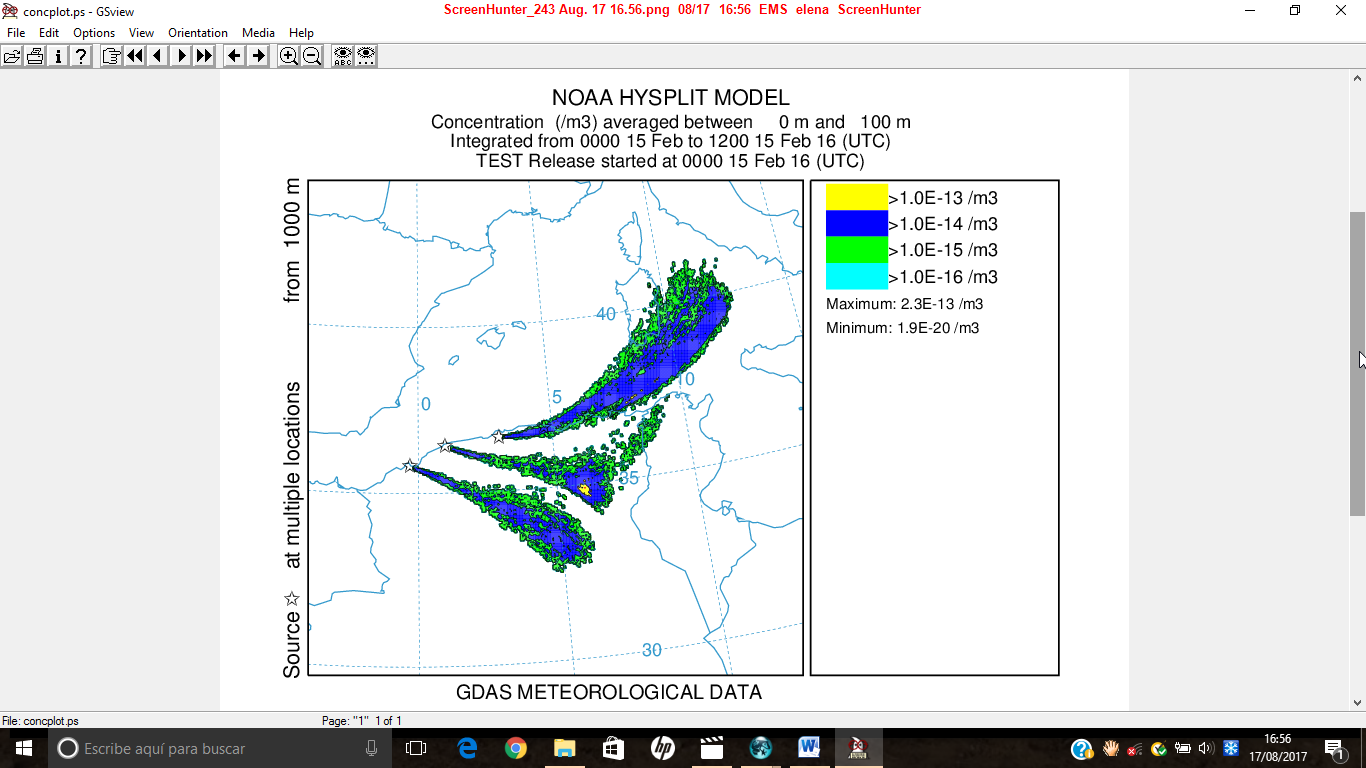

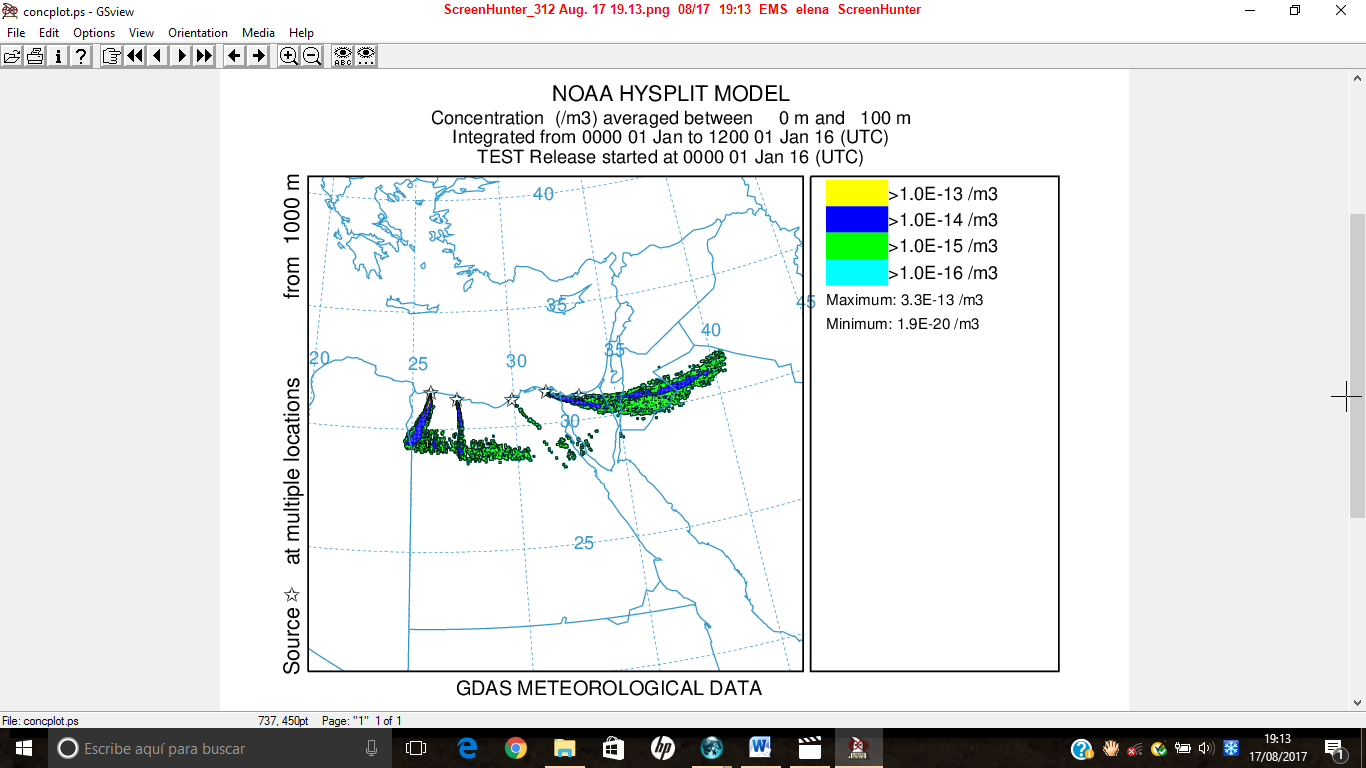

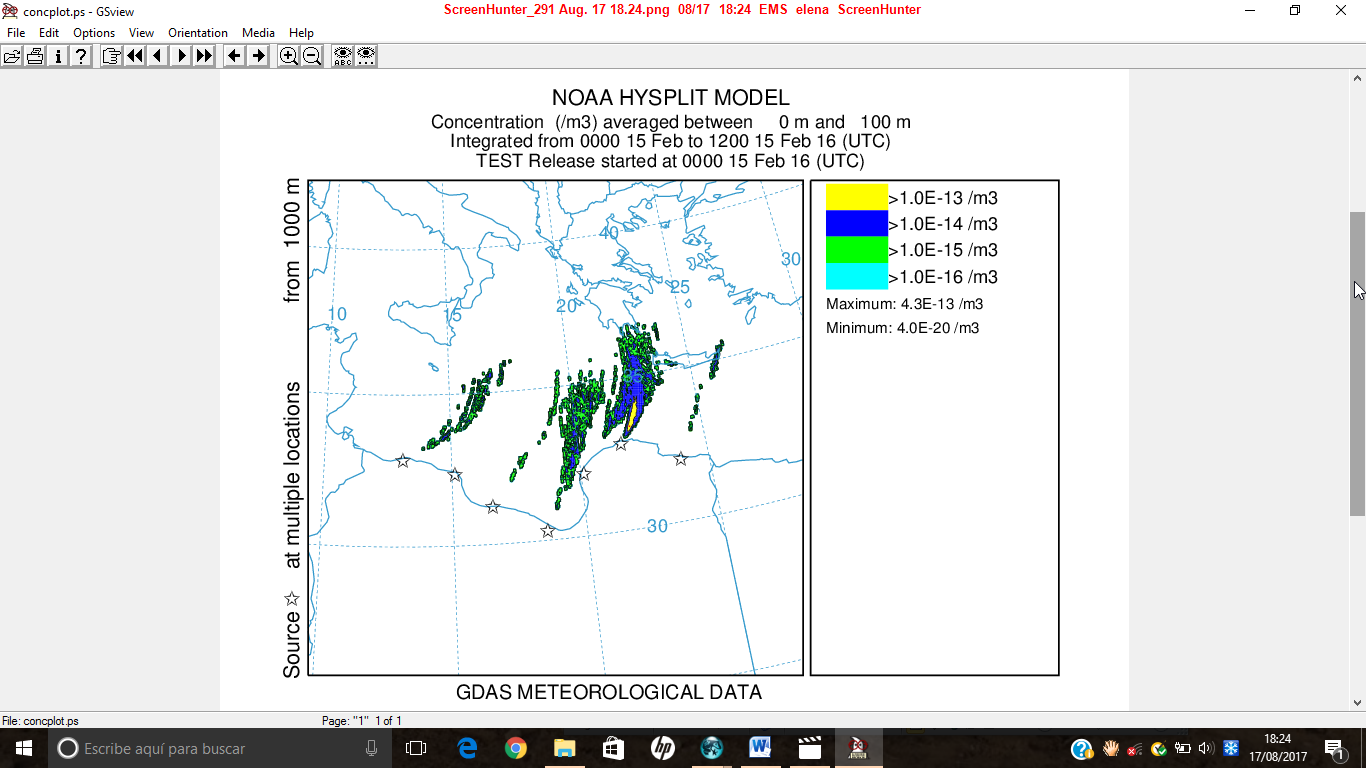

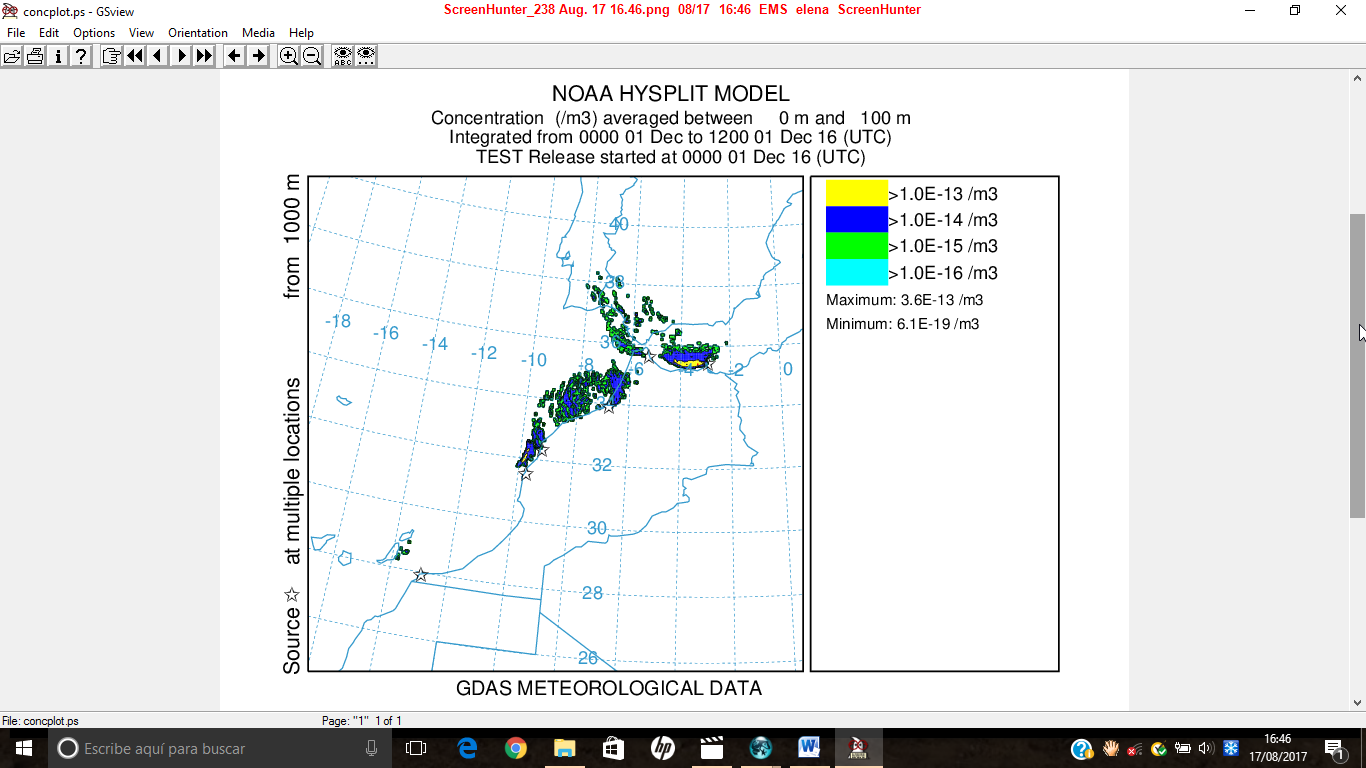

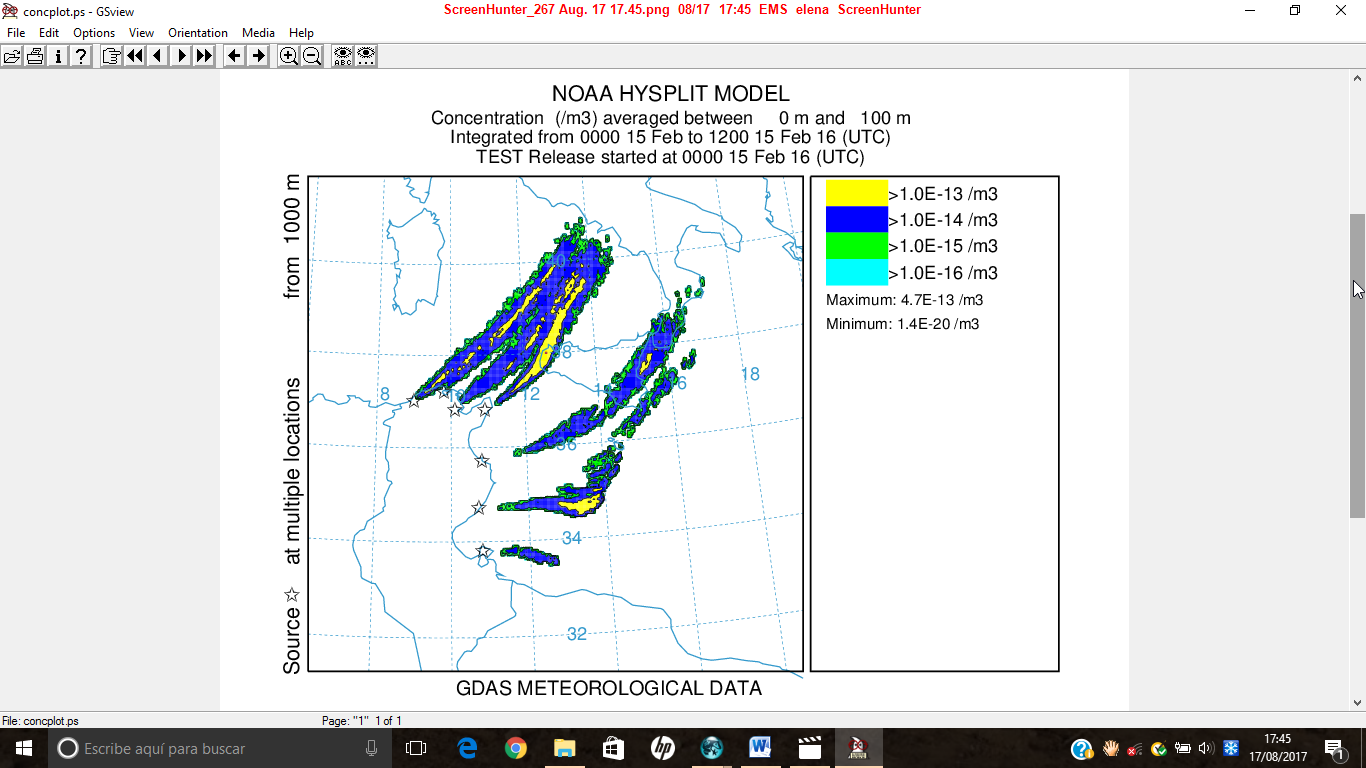

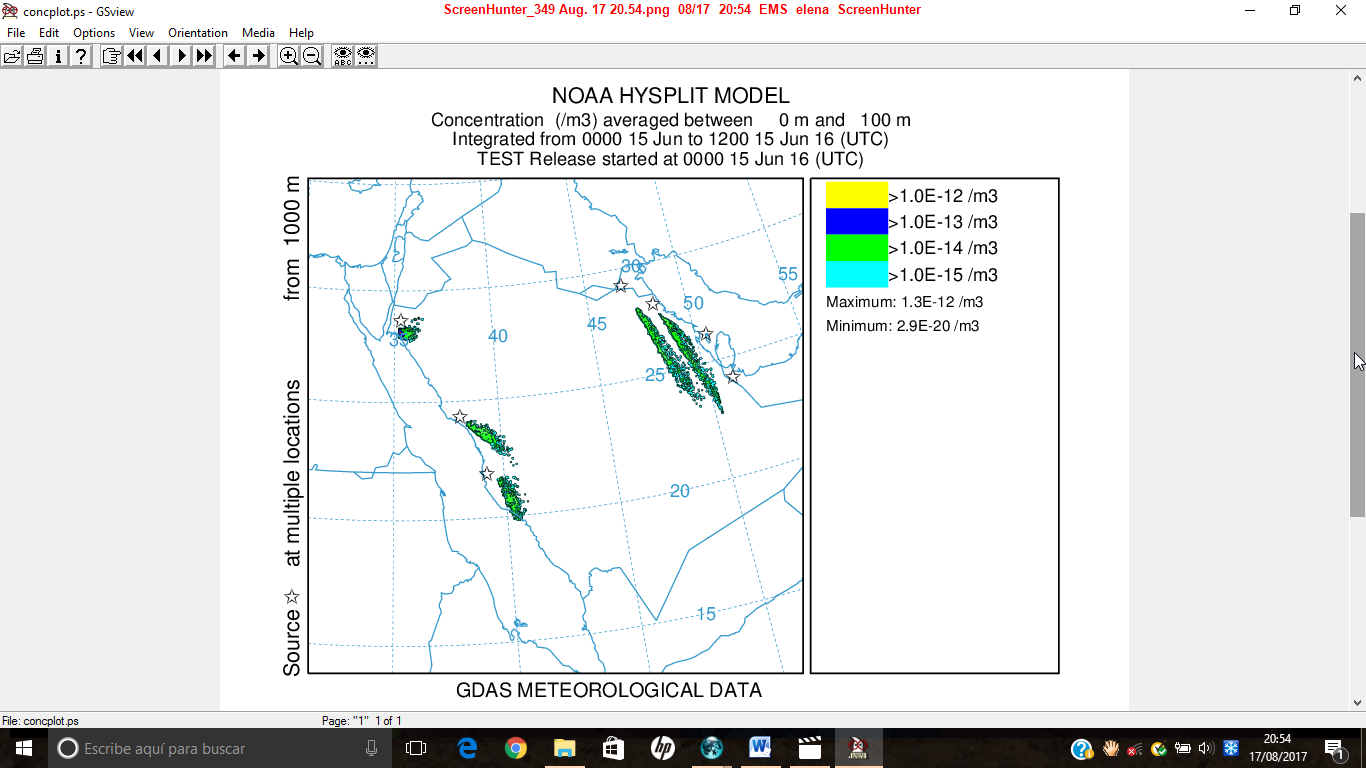


**Supplementary Figure 1.** Collection of different simulations, obtained with HYSPLIT program, of the dispersion of particles in wind currents in different geographical locations.

## Supplementary Tables

**Supplementary Table 1.** Average animal population, expressed in millions, in the countries included on the study in the last two years (OIE World Animal Health Information System).

| **Animal population** | **Country “i”** | | | | | | | | | | | |
| --- | --- | --- | --- | --- | --- | --- | --- | --- | --- | --- | --- | --- |
| **Animal species** | 1 | 2 | 3 | 5 | 6 | 7 | 9 | 11 | 12 | 13 | 15 | 16 |
| Backyard poultry |  |  | 0 |  |  | 34,6 |  |  |  |  |  |  |
| Bees | 0,838 | 1,36 | 0,256 |  |  |  |  |  |  |  |  |  |
| Birds |  |  |  | 649,22 | 46,652 | 15,4 | 36,824 | 129,682 |  | 12 | 42,9 |  |
| Broilers |  | 241,5 | 107,736 |  |  |  | 4,049 |  | 46,92 |  |  |  |
| Buffaloes |  |  |  | 3,51 |  |  |  |  |  |  |  |  |
| Camelidae | 0,395 | 0,36 | 0,056 | 0,099 | 4,792 | 6,4 | 1,018 | 0,21 | 0,118 | 0,002 | 0,392 | 0,252 |
| Cattle | 6,476 | 2,14 | 0,68 | 4,2 | 30,191 | 24,8 | 10,782 | 0,98 | 0,027 | 0,005 | 0,085 | 0,374 |
| Cervidae |  |  |  |  |  |  |  |  |  |  |  | 0,25 |
| Commercial pigs |  |  | 0,002 |  |  |  | 0,083 |  |  |  |  |  |
| Dogs |  |  | 0,553 |  |  |  |  |  |  |  |  |  |
| Equidae | 3,078 | 0,18 | 0,187 | 1,47 | 8,344 | 1,1 | 0,543 | 0,027 | 0,004 | 0,005 | 0,028 | 0,003 |
| Goats | 12,293 | 5,01 | 1,162 | 1,66 | 31,029 | 30,8 | 21,614 | 2,166 | 0,217 | 0,018 | 1,85 | 2,169 |
| Hares/rabnits |  | 2,04 | 0,096 |  |  |  |  |  |  |  |  |  |
| Other commercial pultry |  | 6,93 | 10,176 |  |  |  |  |  | 0,487 |  |  |  |
| Rabbits |  |  |  | 1,89 |  |  |  |  |  |  |  |  |
| Sheep | 38,46 | 28,11 | 6,49 | 3,48 | 39,846 | 26,5 | 15,521 | 12,504 | 0,871 | 0,027 | 2,082 | 0,57 |
| Swine |  |  |  |  |  | 1,7 | 0,082 |  |  |  |  |  |
| Total | 61,54 | 287,63 | 127,394 | 665,529 | 160,854 | 141,3 | 90,516 | 145,569 | 48,644 | 12,057 | 47,337 | 3,618 |

**Supplementary Table 2.** Life animal annual export, expressed in number of heads, from the countries included on the study to the European Union (FAOSTAT and CITES Trade Database).

| **Animal movement** | **Country “i”** | | | | | | | | | | |
| --- | --- | --- | --- | --- | --- | --- | --- | --- | --- | --- | --- |
| **Animal species** | **1** | **2** | **3** | **5** | **9** | **11** | **12** | **13** | **14** | **15** | **16** |
| Backyard poultry | 1041 |  |  |  |  |  |  |  |  |  |  |
| Birds |  |  |  | 2 | 2 | 4 | 7 |  | 13 | 0 | 5 |
| Cattle |  |  | 150 |  |  | 50 |  |  |  | 1265 | 1 |
| Equidae | 64 | 13 | 17 | 4 |  | 8 | 6 | 12 | 64 | 204 | 12 |
| Total | 1105 | 13 | 167 | 6 | 2 | 62 | 13 | 12 | 77 | 1469 | 18 |

**Supplementary Table 3.** Annual export of animal products, expressed in tones, from the countries included on the study to the European Union (FAOSTAT).

| **Animal products movement** | **Country “i”** | | | | | | | | |
| --- | --- | --- | --- | --- | --- | --- | --- | --- | --- |
| **Products** | **1** | **2** | **3** | **5** | **8** | **10** | **11** | **12** | **13** |
| Avian meat |  | 245 | 57 | 5 |  |  |  | 85 | 3 |
| Bovine meat |  |  |  | 1 |  |  |  | 1 | 1 |
| Other meat |  |  | 6 |  |  | 2150 |  |  |  |
| Ovine meat |  |  |  |  |  |  |  |  | 9 |
| Porcine meat |  |  |  |  |  |  |  |  | 1 |
| Food waste |  |  |  | 96 |  |  |  |  |  |
| Fat |  |  |  | 16 |  |  |  |  |  |
| Milk |  |  |  |  | 236 |  |  |  | 2 |
| Cheese | 40 |  |  | 24 |  |  | 80 | 9 |  |
| Bovine offal |  |  |  | 1 |  |  |  |  |  |
| Total | 40 | 245 | 63 | 143 | 236 | 2150 | 80 | 95 | 16 |

**Supplementary Table 4.** International passenger air and maritime transport, expressed in thousands of passengers, from the countries included in the study to the European Union in 2016 (EUROSTAT).

| **Country “i”** | **Number of passengers (thousands)** | |
| --- | --- | --- |
|  | Air transport | Maritime transport |
| 1 | 4948.308 | 2147 |
| 2 | 5342.443 | 350 |
| 3 | 1052.807 | 301 |
| 4 | 290 |  |
| 5 | 3041.062 |  |
| 6 | 218 |  |
| 7 | 40.224 |  |
| 8 | 17 |  |
| 9 | 6 |  |
| 10 | 160 |  |
| 11 | 1227.232 |  |
| 12 | 761.994 |  |
| 13 | 17.993 |  |
| 14 | 172.147 |  |
| 15 | 571.691 |  |
| 16 | 143.457 |  |
